# Production of Biomass-Degrading Multienzyme Complexes under Solid-State Fermentation of Soybean Meal Using a Bioreactor

**DOI:** 10.1155/2012/248983

**Published:** 2012-12-29

**Authors:** Gabriela L. Vitcosque, Rafael F. Fonseca, Ursula Fabiola Rodríguez-Zúñiga, Victor Bertucci Neto, Sonia Couri, Cristiane S. Farinas

**Affiliations:** ^1^Embrapa Instrumentação, Rua XV de Novembro 1452, 13560-970 São Carlos, SP, Brazil; ^2^Instituto Federal de Educação, Ciência e Tecnologia do Rio de Janeiro, Rua Senador Furtado 121, Maracanã 20270-021, RJ, Brazil

## Abstract

Biomass-degrading enzymes are one of the most costly inputs affecting the economic viability of the biochemical route for biomass conversion into biofuels. This work evaluates the effects of operational conditions on biomass-degrading multienzyme production by a selected strain of *Aspergillus niger*. The fungus was cultivated under solid-state fermentation (SSF) of soybean meal, using an instrumented lab-scale bioreactor equipped with an on-line automated monitoring and control system. The effects of air flow rate, inlet air relative humidity, and initial substrate moisture content on multienzyme (FPase, endoglucanase, and xylanase) production were evaluated using a statistical design methodology. Highest production of FPase (0.55 IU/g), endoglucanase (35.1 IU/g), and xylanase (47.7 IU/g) was achieved using an initial substrate moisture content of 84%, an inlet air humidity of 70%, and a flow rate of 24 mL/min. The enzymatic complex was then used to hydrolyze a lignocellulosic biomass, releasing 4.4 g/L of glucose after 36 hours of saccharification of 50 g/L pretreated sugar cane bagasse. These results demonstrate the potential application of enzymes produced under SSF, thus contributing to generate the necessary technological advances to increase the efficiency of the use of biomass as a renewable energy source.

## 1. Introduction

Biomass-degrading enzymes are one of the most costly inputs affecting the economic viability of the biochemical route for biomass conversion into biofuels. This is due to the large scale of the processes involved in biofuel production, and the considerable quantities of enzymes that are required. In addition to quantity, the quality of the enzymatic complex is an important issue, since a cocktail containing cellulases, hemicellulases, pectinases, and other accessory enzymes, acting in synergy in the degradation process, is necessary due to the high recalcitrance of plant biomass. This enzymatic complex is produced by a wide variety of microorganisms (bacteria and fungi); however, the aerobic fungi are known for their higher growth and protein secretion rates [1, 2]. Most commercial cellulases are produced by filamentous fungi of the genera *Trichoderma* and *Aspergillus* [3].

The use of solid-state fermentation (SSF) is particularly advantageous for enzyme production by filamentous fungi, since it simulates the natural habitat of the microorganisms [4]. From the environmental point of view, the main benefit of SSF is the ability to use agroindustrial waste (sugarcane bagasse, wheat bran, soybean meal, etc.) as a solid substrate that acts as a source of both carbon and energy [5]. However, certain operational limitations of SSF, such as difficulty in controlling the moisture level of the substrate, and avoiding heat build-up, have held back its industrial application. Previous studies have shown the importance of evaluating the influence of process operational parameters on cellulase production by SSF, using controlled conditions of forced aeration and inlet air relative humidity [6].

Brazil is currently the second largest producer of soybeans, after the USA. In the 2009/2010 season, the crop occupied an area of 23.6 million hectares and achieved a production of 68.7 million tons [7]. Compared to other crops, soybeans are the third most heavily traded crop in the world. As demand continues to grow, both production areas and trade are likely to increase more rapidly for soybeans than for most other major crops [8]. Soybean meal, the by-product remaining after the extraction of oil from whole soybeans, consists of 44% crude protein, 3.0% crude fiber, 0.5% fat, and 12% moisture [9]. Given its protein-rich composition, this agricultural by-product has considerable potential as a substrate for fungal growth under SSF.

Studies concerning the selection of cultivation conditions for enzyme production by SSF of soybean meal have been described in the literature. The enzymes considered include xylanase [10–12] and cellulase [13, 14], amongst others [15–18]. However, all these studies have been carried out under static cultivation conditions. Therefore, there is great interest in the development of biomass-degrading enzyme production processes using SSF of soybean meal under controlled conditions of forced aeration and inlet air relative humidity.

The present work investigates the effects of operational conditions on the production of biomass-degrading multienzyme complexes (containing FPase, endoglucanase, and xylanase) by a selected strain of *Aspergillus niger*, cultivated under SSF of soybean meal using an instrumented lab-scale bioreactor. Statistical experimental design, with response surface analysis, was used to study the influence of air flow rate, inlet air relative humidity, and initial substrate moisture content on the efficiency of multienzyme production. The enzymatic complex produced under optimized conditions was used to hydrolyze a lignocellulosic biomass (pretreated sugar cane bagasse).

## 2. Materials and Methods

### 2.1. Instrumented Bioreactor

The bioreactor used in the fermentations was a lab-scale system adapted from [19], consisting of 16 columns (2.5 cm diameter, 20 cm length) placed in a water bath. The bioreactor was equipped with an on-line system to control the air flow rate and the inlet air relative humidity, whose description and schematic diagram have been previously reported [6].

### 2.2. Microorganism

The microorganism used in this study was a strain of *A. niger* (known as *A. niger* 12), from the Embrapa Food Technology collection (Rio de Janeiro, Brazil), which had been isolated from black pepper [20]. The culture was maintained in PDA slants at 32°C for 5 days before inoculation. 

### 2.3. SSF Cultivation Conditions

Fermentations were carried out for 72 hours at 32°C, using soybean meal as solid substrate, with a moisture level varying from 56 to 84%, according to the experimental design conditions described in Section 2.4. The moisture content was adjusted with a solution of 0.9% (w/v) ammonium sulfate in 0.1 mol/L HCl. The solid medium was sterilized by autoclaving at 121°C for 20 minutes before inoculation. A spore suspension volume corresponding to 10^7^ conidia/g of dry solid medium was inoculated into the solid medium by gently stirring with a glass rod until a uniform mixture was obtained. The air flow rate and inlet air relative humidity were varied in the ranges 12–36 mL/min and 56–84%, respectively, according to the experimental design described in Section 2.4. 

After the cultivation period, the solid medium was transferred to Erlenmeyer flasks, and the enzymes were extracted by adding a sufficient volume of 0.2 mol/L sodium acetate buffer, at pH 4.5, to achieve a solid/liquid ratio of 1 : 5. The suspension was stirred at 120 rpm for 30 minutes at 32°C, and the enzymatic solution was recovered by filtration. The enzyme extracts were stored at −18°C prior to the analyses.

### 2.4. Experimental Designs

A full factorial design was initially used to evaluate the effects of air flow rate, inlet air relative humidity, and initial substrate moisture content on the efficiency of multienzyme production (as FPase, endoglucanase, and xylanase activities). The experimental design selected was a 2^3^ full factorial design comprising eleven runs, corresponding to eight axial points and three central points, with the experiments carried out in random order. Values of the independent variables and their coded levels are given in Table 1. The significant parameters identified by the full factorial design were then optimized using a response surface methodology (RSM). The central composite design (CCD) used consisted of eleven runs, corresponding to four cube points, four axial points, and three central points (Table 3). The response variables were the enzymatic activities of FPase, endoglucanase, and xylanase. The Statsoft (v. 7.0) statistical software package was used for analysis of the experimental data, application of ANOVA (analysis of variance), and generation of the response surfaces. A second-order polynomial model was used to fit the data:

(1) $$\begin{array}{c} Y\;=\;\beta_{0}+\beta_{1}X_{1}+\beta_{2}X_{2}+\beta_{11}X_{1}^{2}+\beta_{22}X_{2}^{2}+\beta_{12}X_{1}X_{2}, \end{array}$$

where *Y* is the predicted response for enzymatic activity, expressed as IU/g; *β*_0_ is the intercept term; *β*_1_ and *β*_2_ are the linear coefficients; *β*_11_ and *β*_22_ are the squared coefficients; *β*_12_ is the interaction coefficient; and *X*_1_ and *X*_2_ are the coded independent variables. The terms that were not statistically significant were removed from the model and added to the lack of fit.

### 2.5. Multienzyme Production Profile

The multienzyme production efficiency was evaluated during a 96-hour cultivation period, using the operational conditions selected in the experimental design (air flow rate of 24 mL/min, inlet air relative humidity of 70%, and initial substrate moisture content of 84%). Samples were withdrawn at 24-hour intervals, and the enzymes were extracted and analyzed as described in Section 2.7. A respirometric analysis was carried out by measuring CO_2_ in the outlet air stream, using a GMM 220 instrument (Vaisala, Finland). The cumulative amount of CO_2_ produced was calculated from the area under the CO_2_ versus cultivation time curve.

### 2.6. Hydrolysis Experiments

Crude enzymatic extracts, produced under the optimized conditions, were used to hydrolyze a lignocellulosic biomass (steam-exploded sugarcane bagasse, donated by a local sugarcane mill and characterized according to [21]). The pretreated bagasse was washed, dried at ambient temperature, milled, and sieved to obtain a particle size of <1 mm. The enzymatic preparations were diluted in pH 4.8 citrate buffer, and the enzymes were loaded at a rate of 5 FPU/g of dry material. The experiments were carried out for 36 hours at 50°C, in 125 mL Erlenmeyer flasks containing 1.5 g of bagasse and a total liquid volume of 30 mL, with 200 rpm agitation. Samples were collected after defined time intervals, and the concentrations of glucose, xylose, and cellobiose were determined using HPLC [21]. Total reducing groups were quantified according to the DNS method developed by Miller [22]. 

### 2.7. Multienzyme Activity Assays

The activities of FPase and endoglucanase were measured according to the methodology described by Ghose [23]. Here, one unit of activity corresponds to 1 *μ*mol of glucose released per minute, at pH 4.8 and 50°C. Xylanase activity was measured by the method described by Bailey and Poutanen [24]. One unit of xylanase activity corresponds to 1 *μ*mol of xylose released per minute, at pH 5.0 and 50°C. The results were expressed as activity units per mass of initial dry solid substrate (IU/g). 

## 3. Results and Discussion

### 3.1. Influence of SSF Operational Conditions on Multienzyme Production

Biomass-degrading enzymes are present in the form of multienzyme systems whose components have a synergistic action during degradation of the polymeric chains of lignocellulosic materials. Here, a 2³ full factorial design was used first to determine the effects of inlet air relative humidity, air flow rate, and initial substrate moisture content on the efficiency of multienzyme production by *A. niger* grown on soybean meal under SSF. Table 1 presents the experimental conditions and the responses for FPase, endoglucanase, and xylanase production. FPase activity values ranged from 0.13 (run 4) to 0.23 IU/g (runs 5, 6, and 7), endoglucanase activity ranged from 27.5 (run 3) to 42.6 IU/g (run 10), and xylanase activity ranged from 47.9 (run 2) to 53.7 IU/g (run 10).

The data were analyzed to determine the effect of each variable (Table 2). Each enzyme showed different behavior in terms of the influence of the operational conditions. All three independent variables showed a statistically significant influence on FPase activity, within a confidence limit of 90%. Inlet air relative humidity and air flow rate showed negative effects, while initial substrate moisture content showed a positive effect on FPase production, within the range tested. For endoglucanase, both inlet air relative humidity and initial substrate moisture content showed significant positive influences, within a confidence limit of 95%. None of the variables showed any significant influence on xylanase production, within the ranges tested.

Based on the statistical results, a new factorial design was drawn up in order to implement the optimization of the variables. The ranges of the inlet air relative humidity and initial substrate moisture content were expanded to 56–84%, taking into consideration the saturation limit of the substrate. Since the air flow rate showed no significant effect on the activity of either endoglucanase or xylanase, within the range tested, this variable was fixed at the central point value used previously (24 mL/min).

### 3.2. Optimization of Multienzyme Production

The significant parameters identified using the 2³ full factorial design were further optimized using a response surface methodology (RSM). In this procedure, the effects of inlet air relative humidity and initial substrate moisture content on multienzyme production efficiency were studied using a central composite design (CCD). Table 3 presents the experimental conditions and the responses for FPase, endoglucanase, and xylanase production. FPase activity values ranged from 0.07 (run 5) to 0.22 IU/g (runs 3 and 4), endoglucanase activity ranged from 30.2 (runs 2 and 7) to 39.3 IU/g (run 8), and xylanase activity ranged from 30.9 (run 6) to 51.1 IU/g (run 4).

**Figure 1:**
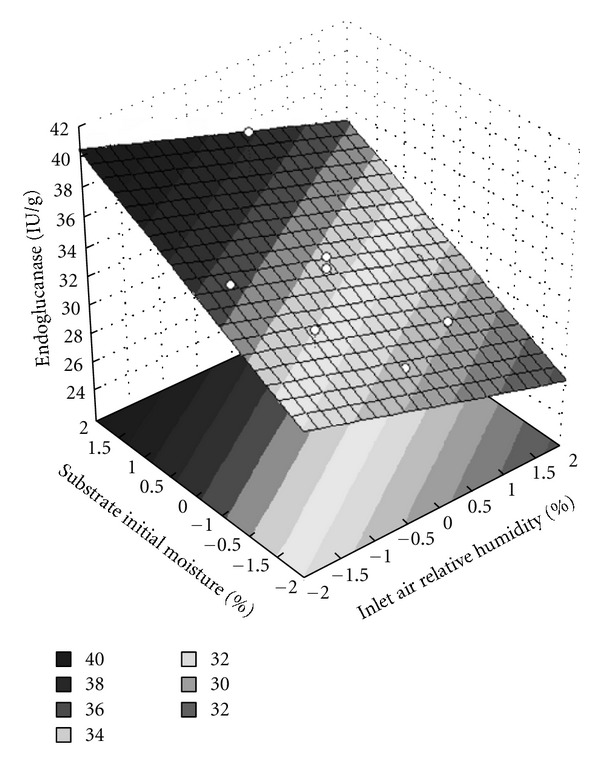
Response surface plot for the effects of initial substrate moisture content and inlet air relative humidity on endoglucanase activity.

Application of analysis of variance (ANOVA) to the multienzyme production results (Table 4) gave a correlation coefficient of 0.7748 and an *F* value of 13.76 (3.09-fold higher than the listed *F* value, at a 95% confidence level) for endoglucanase activity. These values were satisfactory for prediction using the model ((1), with the coefficients listed in Table 4) employed to describe the response surface plot for endoglucanase production (Figure 1). For FPase and xylanase, it was not possible to obtain a quadratic model that represented the process, since the *F* values and correlation coefficients were both low in the case of these enzymes.

At a fixed air flow rate of 24 mL/min, higher endoglucanase production (39.3 IU/g) by *A. niger* was obtained using an inlet air relative humidity of 70% and an initial substrate moisture content of 84%. Under these conditions, the activities of FPase and xylanase were 0.20 and 41.6 IU/g, respectively. In previous work, endoglucanase production of up to 56.1 IU/g was achieved using wheat bran as solid substrate [6]. Even though lower endoglucanase production was obtained in the present study, both values are of the same order of magnitude. A comparison between the composition of soybean meal and wheat bran is presented in Table 5. Although soybean meal has a higher protein and cellulose content than wheat bran, other characteristics such as higher lignin content and differences in their porosity can be contributing to the lower enzymatic production values achieved by *A. niger* cultivated in soybean meal.

It is interesting to note that the optimization using CCD did not result in higher multienzyme production values, compared to the full factorial design, indicating that the conditions used in the first experimental design were close to the optimum values. Nevertheless, using CCD it was possible to obtain a quadratic model that represented endoglucanase production and the influence of both inlet air relative humidity and initial substrate moisture content on the SSF process.

The initial substrate moisture content, as well as the aeration rate, was shown by Spier et al. [39] to exert a significant influence on phytase production by *A. niger* cultivated in a column-type SSF bioreactor. Among the various operational parameters that affect SSF process efficiency, moisture content is one of the most important. If the moisture content is too high, the void spaces in the solids are filled with water, resulting in oxygen limitation. At the other extreme, if the moisture content is too low, the growth of the microorganism will be hindered [40]. Consequently, identification of the optimal moisture content for each solid substrate is crucial for the promotion of favorable growing conditions, and hence for satisfactory metabolite production. However, the optimal moisture content value depends on both the solid substrate and the microorganism used [5]. 

The effect of the initial substrate moisture content on the production of cellulase using SSF has also been described previously. Mamma et al. [32] evaluated enzyme production under SSF, using the fungus *A. niger* with orange peel as substrate, and were able to significantly increase enzyme activities after optimizing the initial moisture content of the solid medium. Gao et al. [25] found that an increase in the initial moisture content enhanced enzyme production by the thermoacidophilic fungus *Aspergillus terreus* M11, cultivated under SSF using corn stover as substrate.

### 3.3. Multienzyme Production Profiles under Selected Conditions

**Figure 2:**
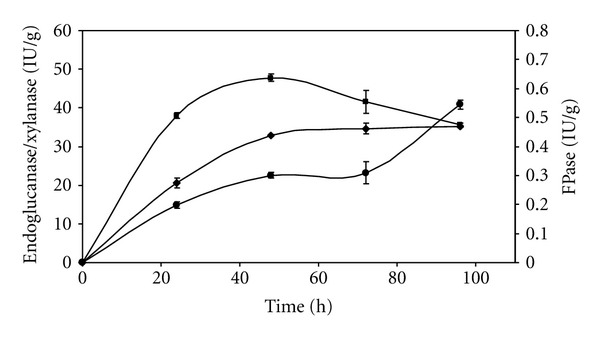
Kinetics of multienzyme production during *A. niger *cultivation in soybean meal at 84% initial moisture content, with a flow rate of 24 mL/min and an inlet air relative humidity of 70%. (●) FPase, (♦) endoglucanase, (▪) xylanase.

The profiles of multienzyme production over a period of 96 hours, using an initial substrate moisture content of 84%, an inlet air humidity of 70%, and a flow rate of 24 mL/min are illustrated in Figure 2. Xylanase production reached its highest value (47.7 IU/g) after 48 hours of cultivation, whereas the highest values for endoglucanase and FPase (35.1 and 0.55 IU/g, resp.) were only achieved after around 96 hours. The profiles of xylanase and cellulase production during cultivation therefore appeared to be influenced by the presence of the lignocellulosic biomass, with initial production of xylanases in order to degrade the hemicellulosic fraction, followed by production of cellulases for the conversion of cellulose to sugars.

**Figure 3:**
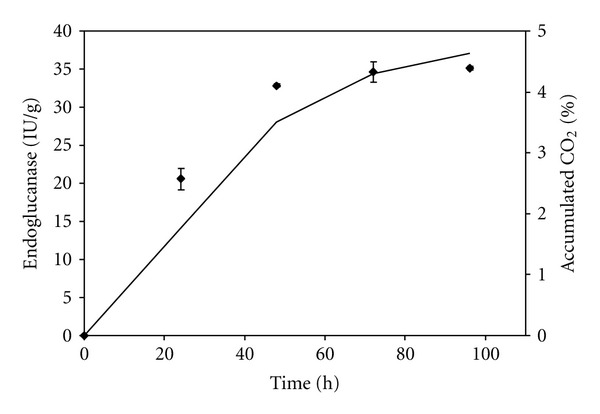
Endoglucanase production kinetics and cumulative CO_2_ production (full line, left *y* axis) during *A. niger *cultivation in soybean meal at 84% initial moisture content, with a flow rate of 24 mL/min and an inlet air relative humidity of 70%.

The evolution of CO_2_ during the SSF process was monitored using a sensor connected to the gas stream exiting the columns. CO_2_ data can provide an important means of understanding the relationship between fungal growth and enzyme production, since it is difficult to measure biomass in SSF due to the problem of separating the biomass from the substrate [5]. A comparison between endoglucanase production and the cumulative evolution of CO_2_ during cultivation of *A. niger* is shown in Figure 3. The conditions used were an inlet air relative humidity of 70%, an air flow rate of 24 mL/min, and an initial substrate moisture content of 84%. CO_2_ production correlated well with endoglucanase production (*R* ^2^ = 0.9448). A similar result was obtained in our earlier study of endoglucanase production using wheat bran as solid substrate [6]. It was not possible to obtain any correlations between the cumulative evolution of CO_2_ and either FPase or xylanase.

The comparisons of cellulase and xylanase activities produced under SSF by other *Aspergillus* strains showed that the results obtained in this work compared favorably with those reported in the literature, although there are a number of superior activity values, specially for xylanase activity (Table 6). However, it should be highlighted that this work was not optimized in terms of xylanase, with this enzyme being usually produced simultaneously by organisms with cellulolytic activities. The sources of variability that should be taken into consideration when analyzing the data presented in Table 6 include the characteristics of the fungal strain (mutation, thermophilicity, and others) as well as the differences in cultivation conditions such as moisture content, temperature, incubation period, and substrate used for SSF. It was also observed that cellulase and xylanase activity assays can vary considerably among laboratories (including substrate, reaction temperature, pH, and time). Therefore, it is difficult to compare yields of enzymes produced by SSF, but values given in Table 6 provide a notion about their order of magnitude and should be used only as a guideline for comparing the different systems reported.

### 3.4. Hydrolysis Experiments

A set of enzymatic hydrolysis experiments using pretreated sugarcane bagasse were conducted in order to determine the hydrolytic potential of the multienzyme complex produced by *A. niger* cultivated under the optimized conditions identified in the CCD procedure. 

**Figure 4:**
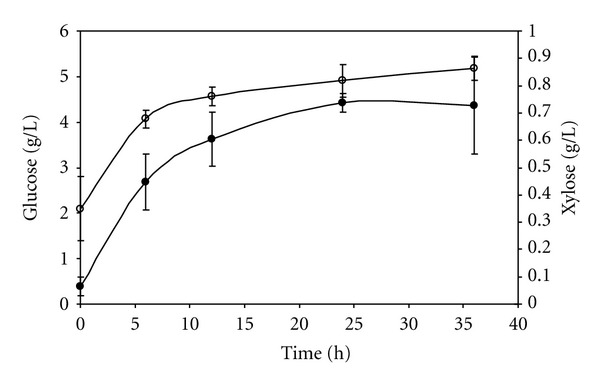
Hydrolysis of 5% (w/v) steam-pretreated sugarcane bagasse at pH 4.8 and 50°C, using enzyme cocktails produced during *A. niger *cultivation in soybean meal at 84% initial moisture content, with a flow rate of 24 mL/min and an inlet air relative humidity of 70%. (●) Glucose, (*⚪*) xylose.

The enzyme loading corresponded to 5 FPU/g of cellulose.  Figure 4 illustrates the temporal profiles of the concentrations of glucose and xylose, during saccharification of the pretreated bagasse. Cellobiose was also analyzed, but was not detectable, indicating that there was no accumulation of this compound, probably due to the presence of *β*-glucosidase in the enzymatic complex. The amount of glucose released in 36 hours of saccharification was 4.4 g/L. In terms of the glucose yield after 36 hours of hydrolysis, calculated from the cellulose content of the pretreated bagasse, this value corresponds to a conversion rate of 14.2%.

The nonlinear profile observed for the hydrolysis of the pretreated bagasse was expected due to the heterogeneous nature of the lignocellulosic biomass. As a result, extension of the hydrolysis time beyond 24 hours showed little additional increment in the hydrolysis yield. Such behavior can be explained by the presence of lignin in the pretreated bagasse (Table 5). It has been reported that the presence of lignin impedes the performance of the enzyme during hydrolysis by creating nonproductive enzyme-lignin bonds [41]. Thus, a partial delignification of the pretreated material before enzymatic hydrolysis could possibly lead to higher sugar yields.

The profile of xylose release from pretreated bagasse was also nonlinear (Figure 4), with 0.86 g/L being released after 36 hours of hydrolysis. Considering that there was a small concentration of xylose (0.35 g/L) in the initial hydrolysis supernatant, which was probably already present in the enzymatic extract, the amount of xylose released was relatively low. Such low concentration of xylose can be explained by the partial removal of the hemicellulosic fraction in the pretreated sugarcane bagasse used (Table 5). Steam explosion, which has been considered a potential pretreatment technology for sugarcane bagasse, extracts the more soluble polymers preventing their subsequent hydrolysis [42].

Gottschalk et al. [43] produced an enzymatic cocktail using *A. awamori* cultivated under submerged fermentation. Hydrolysis of pretreated sugarcane bagasse using the enzymes produced, at a loading of 10 FPU/g of solids, resulted in the formation of 3.5 g/L of glucose within 72 hours, similar to the value of 4.4 g/L obtained here. Overall, the results of the hydrolysis experiments indicated that the enzymatic cocktail produced by *A. niger* has good potential for use in the conversion of biomass.

## 4. Conclusions

The influence of SSF process variables on fungal growth and biomass-degrading multienzyme production was studied using a lab-scale instrumented bioreactor. The results obtained enabled selection of the variables that could be adjusted in order to improve multienzyme production. Highest enzymatic activities were obtained using an initial substrate moisture content of 84%, an inlet air humidity of 70%, and an air flow rate of 24 mL/min. The enzymatic complex produced under the optimized conditions was used to hydrolyze a lignocellulosic biomass, releasing 4.4 g/L of glucose after 36 hours of saccharification of 50 g/L pretreated sugar cane bagasse. The enzymatic complex therefore showed good potential for use in biomass conversion.

## Figures and Tables

**Table 1:** Full factorial design for multienzyme production under different operational conditions.

Run	Levels			Responses		
	Inlet air relative humidity (%)	Flow rate (mL/min)	Substrate initial moisture (%)	FPase (IU/g)	Endoglucanase (IU/g)	Xylanase (IU/g)
1	−1 (60)	−1 (12)	−1 (60)	0.21	29.9	48.8
2	1 (80)	−1 (12)	−1 (60)	0.19	32.8	47.9
3	−1 (60)	1 (36)	−1 (60)	0.20	27.5	48.2
4	1 (80)	1 (36)	−1 (60)	0.13	30.1	44.6
5	−1 (60)	−1 (12)	1 (80)	0.23	31.2	49.3
6	1 (80)	−1 (12)	1 (80)	0.23	37.5	48.9
7	−1 (60)	1 (36)	1 (80)	0.23	30.7	50.7
8	1 (80)	1 (36)	1 (80)	0.16	38.0	50.6
9	0 (70)	0 (24)	0 (70)	0.21	40.7	51.8
10	0 (70)	0 (24)	0 (70)	0.17	42.6	53.7
11	0 (70)	0 (24)	0 (70)	0.18	40.8	51.2

**Table 2:** Effects of independent variables on multienzyme activity, based on 2^3^ full factorial design experiments.

	FPase		Endoglucanase		Xylanase	
	Effect	*P* value	Effect	*P* value	Effect	*P* value
Mean	0.195**	0.000	34.711*	0.000	49.621*	0.000
(1) Inlet air relative humidity	−0.041**	0.033	4.762*	0.023	−1.270	0.293
(2) Flow rate	−0.034**	0.054	−1.263	0.230	−0.175	0.864
(3) Substrate initial moisture	0.033**	0.060	4.259*	0.029	2.487	0.109
1 × 2	−0.030**	0.080	0.198	0.814	−0.601	0.572
1 × 3	0.003	0.801	2.038	0.110	0.996	0.383
2 × 3	−0.003	0.824	1.247	0.234	1.757	0.190
*R*	0.88211		0.34182		0.42923	

*Significant at 0.05 level; **Significant at 0.1 level; *R*: coefficient of determination.

**Table 3:** Central composite design for multienzyme production under different inlet air relative humidity and initial substrate moisture content conditions.

Run	Levels		Enzymes (IU/g)		
	Inlet air relative humidity (%)	Substrate initial moisture (%)	FPase	Endoglu-canase	Xylanase
1	−1 (60)	−1 (60)	0.18	33.9	42.0
2	+1 (80)	−1 (60)	0.15	30.2	42.0
3	−1 (60)	+1 (80)	0.22	34.9	48.6
4	+1 (80)	+1 (80)	0.22	33.4	51.1
5	−1,41 (56)	0 (70)	0.07	35.2	39.2
6	1.41 (84)	0 (70)	0.16	31.2	30.9
7	0 (70)	−1,41 (56)	0.20	30.2	49.8
8	0 (70)	1.41 (84)	0.20	39.3	41.6
9	0 (70)	0 (70)	0.16	34.2	40.9
10	0 (70)	0 (70)	0.13	33.1	38.1
11	0 (70)	0 (70)	0.09	33.4	39.3

**Table 4:** Values of coefficients, and statistical analysis of multienzyme activity, based on central composite design experiments.

	FPase		Endoglucanase		Xylanase	
	Coefficient	*P* value	Coefficient	*P* value	Coefficient	*P* value
Mean	0.13**	0.025	33.56*	0.000	39.39*	0.000
Inlet air humidity *X*_1_ (L)	0.01	0.401	−1.35*	0.022	−1.15	0.155
Inlet air humidity *X*_1_ (Q)	0.00	0.804	−0.39	0.245	−0.79	0.325
Substrate initial moisture *X*_2_ (L)	0.01	0.375	2.14*	0.009	0.53	0.410
Substrate initial moisture *X*_2_ (Q)	0.05**	0.092	0.37	0.268	4.57*	0.017
*X*_1_ (L) × *X*_2_ (L)	0.01	0.708	0.54	0.198	0.66	0.460
*R*	0.61511		0.77479		0.46473	
*F* value	1.60		13.76		0.87	
*F*_cal_/*F*_listed_	0.46		3.09		0.17	

*Significant at 0.05 level; **Significant at 0.1 level; *R*: coefficient of determination.

**Table 5:** Composition of lignocellulosic materials [13].

	Cellulose (%)	Hemicellulose (%)	Lignin (%)	Protein (%)
Soybean meal	34.59	18.13	9.78	43.22
Wheat bran	10.86	28.88	4.89	17.61
Pretreated sugarcane bagasse	61.50	4.51	32.05	—

**Table 6:** Comparison of biomass-degrading enzymes production by *Aspergillus* strains cultivated under SSF.

Organism	Substrate	Incubation time	Xylanase (IU/g)	Endoglucanase (IU/g)	FPAse (IU/g)	Reference
*Aspergillus terreus* M11	Corn stover	96 h	—	563	231	[25]
*A. niger* NS-2	Wheat bran	96 h	—	310	17	[26]
*Aspergillus fumigatus* fresenius	Rice straw	5 days	2800	240.2	9.73	[27]
*A. terreus *	Rice straw	7 days	—	233.7	10.96	[28]
*Aspergillus niger* NRRL-567	Apple pomace	48 h	1412.58	172.31	133.68	[29]
*A. niger* P47C3	Soybean bran	5 days	484.2	152	5.6	[14]
*A*. *niger* MTCC 7956	Wheat bran	72 h	—	135.44	4.55	[30]
*A. niger* KK2	Rice straw	4–6 days	5070	129	19.5	[31]
*A. niger* BTL	Orange peels	6 days	77.1	60.5	—	[32]
*A. niger *	Wheat bran	72 h	—	56.1	—	[6]
*A. niger *	Soybean meal	96 h	47.7	35.1	0.55	This work
*A. niger *	Wheat bran	48 h	170	30	1.13	[33]
*A. nidulans* MTCC344	Sugarcane bagasse	8 days	—	28.96	—	[34]
*A. niger *	Wheat bran	72 h	—	21	0.4	[13]
*A. humus*	Wheat bran and rice straw	5 days	740	12.94	6.28	[35]
*A. niger *	Mango residue	74 h	—	7.26	2.55	[36]
*A. awamori *	Grape pomace and orange peels	10 days	32	5.4	—	[37]
*A. niger* 3T5B8	Mango peel	24 h	50.82	—	8.75	[38]
*A. niger *	Sugarcane bagasse and soybean meal	96 h	3099	—	—	[10]
